# A Meta-Analysis on the Effects of Hydroxychloroquine on COVID-19

**DOI:** 10.7759/cureus.10005

**Published:** 2020-08-24

**Authors:** Nowair Hussain, Emily Chung, Jonathan J Heyl, Bisma Hussain, Michael C Oh, Candis Pinon, Soumya Boral, David Chun, Benson Babu

**Affiliations:** 1 Medicine, St. John's Episcopal Hospital, New York, USA; 2 Medicine, Mount Sinai Hospital, New York, USA; 3 Medicine, Presidency University, Kolkata, IND; 4 Medicine, Northwell Health, New York, USA; 5 Hospital Medicine, Northwell Health, New York, USA

**Keywords:** hydroxychloroquine, chloroquine, azithromycin, covid-19, coronavirus, sars-cov-2

## Abstract

Introduction

Since December 2019, severe acute respiratory syndrome coronavirus 2 (SARS-CoV-2) has rapidly spread throughout the world with a large medical and economic impact. On March 12, 2020, the World Health Organization (WHO) classified SARS-CoV-2 as a pandemic. As a result of this worldwide public health crisis, politicians, elected officials, and healthcare professionals emergently began trialing hydroxychloroquine (HCQ) in efforts to treat and prevent the transmission of the virus. This meta-analysis was performed to assess the effects of HCQ on patients with COVID-19.

Methods

This meta-analysis adheres to the Preferred Reporting Items for Systematic Reviews and Meta-Analyses (PRIMA) guidelines. Selected articles published between December 2019 and July 2020 were found utilizing the following search engines: PubMed, Google Scholar, Cochrane Library, DisasterLit, Clinicaltrials.gov, Medrxiv, and Embase. Two independent physician reviewers screened eligible articles that met the inclusion and exclusion criteria of the analysis. The outcome measures analyzed were mortality rate, rate of disease progression/improvement, rate of disease severity, and adverse effects of treatment. Six out of 14 studies that met the study’s eligibility criteria were selected and further analyzed, with a total of 381 participants (n= 381).

Conclusion

From the studies analyzed, it was found that groups treated with HCQ had an overall mortality rate that was 2.5 times greater than that of the control group. HCQ treated patients had higher rates of adverse clinical outcomes and side effects compared with the control populations. Lastly, there was a 1.2 times higher rate of improvement in the group of HCQ treated patients with mild to moderate symptoms as compared to the control group.

## Introduction

Coronavirus disease (COVID-19) originated in Wuhan, Hubei Province of China on December 15, 2019 [1-4]. The rapid spread of the virus led the World Health Organization (WHO) to announce COVID-19 as a pandemic on March 12, 2020 [5]. The spread of SARS-CoV-2 resulted in an enormous public health crisis with high patient mortality and significant economic consequences [1,6]. Furthermore, COVID-19 is a complex, multifaceted, multi-system disease process that spares no one [7].

The COVID-19 acute respiratory distress syndrome consists of a period of cytokine storm, which is noted particularly in the later stages of advanced severe respiratory failure [8]. COVID-19 patients have increased levels of plasma pro-inflammatory cytokines and chemokines [9-10]. These cytokines and chemokines are IL1b, IL1RA, IL7, IL8, IL9, IL10, basic FGF2, GCSF, GMCSF, IFNg, IP10, MCP1, MIP1a, MIP1b, PDGFB, TNFa, and VEGFA [9-10]. High patient mortality is caused by the disarray of these host cytokines, causing damage to the lungs and leading to multi-system organ failure [10-11].

The SARS-CoV-2 virus has an affinity for the ciliated cells of the respiratory conducting airway, with increased viral replication as it progresses further along the respiratory tract and gastrointestinal mucosa [12]. The SARS CoV-2 infection occurs in three distinct stages: an asymptomatic stage, an upper airway stage, and, finally, the conducting airway response stage, which leads to the classically seen ground-glass infiltrates on chest X-ray and clinical hypoxia with progression to acute respiratory distress syndrome (ARDS) and multi-system dysfunction [13]. In stage 1, the virus binds to the angiotensin-converting enzyme 2 (ACE2) receptor, a transmembrane protease, serine 2 (TMPRSS2). TMPRSS2 is ubiquitous in the human body; it is found in the nasal cavity and lung and is also expressed throughout the intestine and prostate [14]. ACE2 receptors can also be found in the heart, esophagus, kidneys, stomach, bladder, and ileum [12,14]. As SARS-CoV-2 progresses down the respiratory tract, the virus begins to activate a more potent immune response and certain patients may manifest clinically with respiratory failure and ARDS.

Most patients will have a mild disease, with the disease restricted to the upper respiratory tract [13]. About one out of five SAR-CoV-2 infected patients will progress to more severe respiratory disease and further to ARDS [13]. The proposed mechanism is the destruction of type II pneumocytes once the virus reaches the alveoli [12]. The virus would then begin the replication process within these cells and the cell would undergo apoptosis, releasing viral particles. This cellular apoptosis results in diffuse alveolar damage with the formation of hyaline membranes, which decrease gas exchange and lead to clinical hypoxia. Furthermore, the healing of the affected areas may worsen the patient’s condition through more severe parenchymal scarring and fibrosis. Because the cytokines mentioned above have binding sites within the lungs, they may serve as therapeutic targets.

4-Aminoquinolones such as hydroxychloroquine (HCQ) and chloroquine have gained a lot of steam in the medical field and media for their possible efficacy against COVID-19. HCQ has immunomodulatory properties and was originally developed as an antimalarial drug with further applications in patients with rheumatoid arthritis and systemic lupus erythematosus [15]. In vitro studies of HCQ have additionally shown antiviral properties; it supposedly prevents COVID-19 related ARDS [8,15-16]. The treatment of COVID-19 positive patients with HCQ has been met with controversy, as there have been no large multicenter randomized control trials to support its use. Up to this point, there is a lack of statistically significant reduction in morbidity or mortality in COVID-19 patients who have undergone HCQ trials.

The treatment of COVID-19 with a combination of hydroxychloroquine and azithromycin was first proposed in a controversial, small non-randomized trial from the South of France that concluded that the drug combination was effective for the treatment of COVID-19 [17]. Criticism was brought on immediately when it was presented for peer review due to many methodological flaws, with the biggest being the lack of a randomized control group [17]. This led to various expert researchers criticizing the efficacy of hydroxychloroquine, with the majority concluding no statistically significant difference between treatment groups.

The emergent approval of HCQ at the height of the COVID-19 pandemic was considered controversial but necessary given the overwhelming lack of effective treatment options at that time. The controversy was limited not only to the unknown efficacy and side-effect profile of HCQ but also to the limited supply of the drug [18].

## Materials and methods

**Figure 1:**
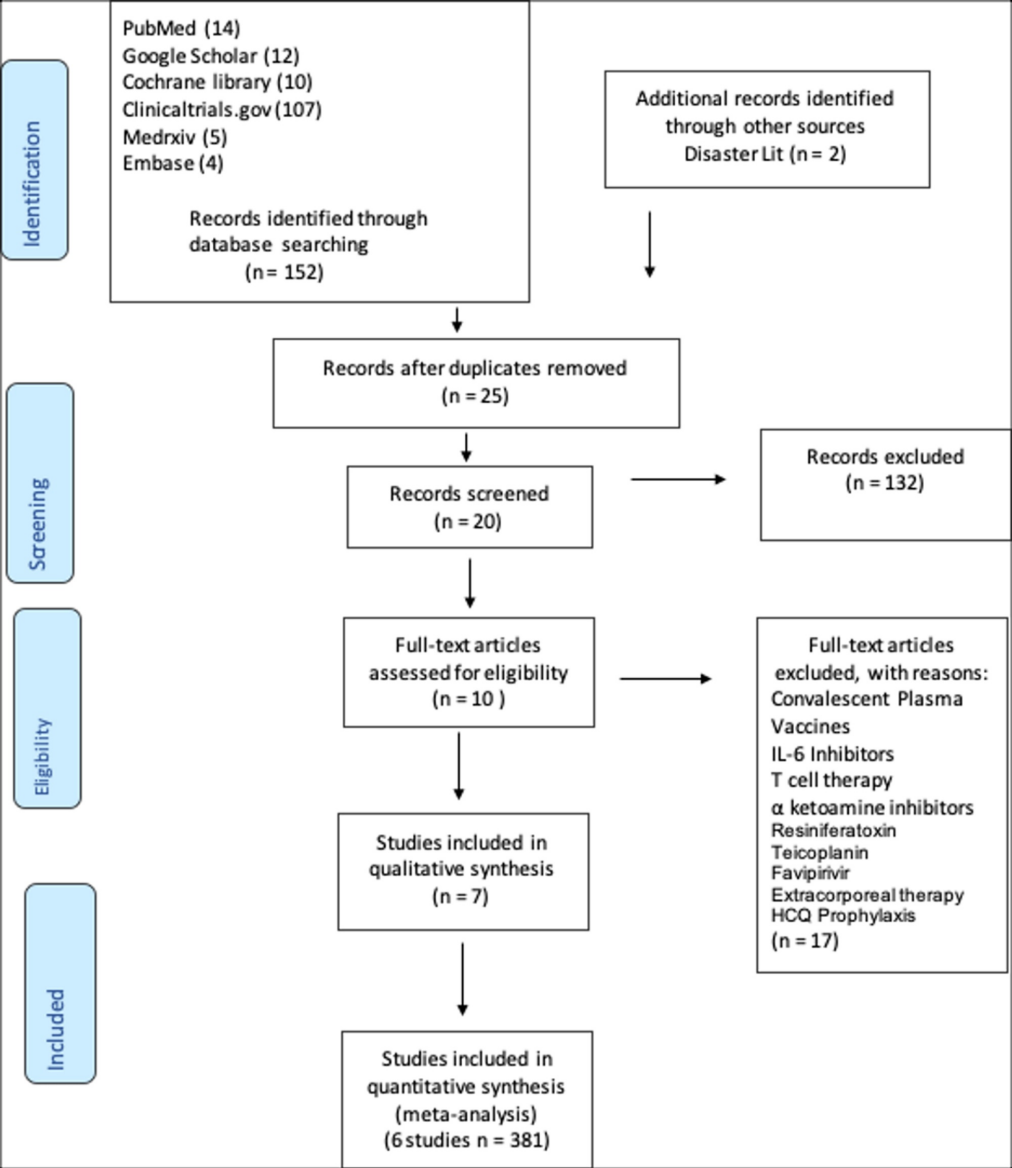
PRISMA flow diagram PRISMA: Preferred Reporting Items for Systematic Reviews and Meta-Analyses

This systematic review and meta-analysis adheres to the Preferred Reporting Items for Systematic Reviews and Meta-Analyses (PRISMA) guidelines (Figure 1) [2]. The search terms used were hydroxychloroquine, chloroquine, azithromycin, COVID-19, coronavirus, and SARS-CoV-2. Using these terms, the systematic search strategies used were boolean and fuzzy logic, truncated terms, and wild cards. Selected articles published between December 2019 and July 2020 were found utilizing the following search engines: PubMed, Google Scholar, Cochrane Library, DisasterLit, Clinicaltrials.gov, Medrxiv, and Embase. Two independent physician reviewers screened eligible articles that met the analysis’ inclusion and exclusion criteria. The inclusion criteria were (1) age range 12-65, (2) prospective control trial, and (3) use of hydroxychloroquine, chloroquine, lopinavir-ritonavir, or azithromycin. The exclusion criteria were (1) presence of a co-morbid medical condition, i.e., advanced heart, liver, or renal disease or diabetes mellitus, (2) treatment with remdesivir, convalescent plasma, corticosteroids, vaccines, IL-6 inhibitors, T-cell therapy, α-ketoamide inhibitors, resiniferatoxin, teicoplanin, favipiravir, extracorporeal therapy, or HCQ prophylaxis.

The outcome measures analyzed were HCQ’s effect on COVID-19 mortality rate, rate of disease progression/rate of improvement, rate of disease severity, and adverse effects of treatment. Six out of 14 studies that met the study’s eligibility criteria were selected and further analyzed, with a total of 381 participants (n= 381).

Data collected from six different studies looked at the effects of HCQ on patients with clinically proven COVID-19 infection. These six studies have been labeled S1, S2, S3, S4, S5, and S6 for better data visualization. Each study varies in sample sizes and the distribution of treatment and control groups. The defined outcomes studied in this meta-analysis are:

1) The mortality rate of patients after applying HCQ on patients with COVID-19

2) The rate of progression/improvement of COVID-19 disease

3) The rate of COVID-19 disease severity, for example, after applying the HCQ treatment, the rate of which patients went on to develop severe conditions such as acute hypoxic respiratory failure and adult respiratory distress syndrome.

The random-effects model was used on the assumption that the study effect estimates show more variance when drawn from a single population [19]. Therefore, this follows the so-called assumption of exchangeability [19]. This means that in a random-effects model fit, not only do assumptions of the effects of individual studies deviate from the true intervention effect of all studies due to sampling error but that there is another source of variance introduced by the fact that the studies do not stem from one single population [19]. The studies are sampled from a “universe” of populations [19]. In this study, the random effect model is a suitable choice because it is a risky assumption to state all the studies along with their respective effect sizes stem from a single homogeneous population.

## Results

Section A: meta-analysis on mortality rates

Among the six studies considered for meta-analysis, information on mortality rates was available in two of them, details of which are provided below (Tables 1-3).

**Table 1:** Studies used in the meta-analysis of mortality rate

Name	Author	Sample Size	num_ control	num_ treatment	mortality_ control	mortality_ treatment
A pilot study of hydroxychloroquine in the treatment of patients with common coronavirus disease-19 (COVID-19)	Chen J, et al. [20]	30	15	15	0	0
Effect of high VS low doses of chloroquine diphosphate as adjunctive therapy for patients hospitalized with severe acute respiratory syndrome coronavirus 2 (SARS-CoV-2) infection: a randomized clinical trial.	Borba, Mayla Gabriela Silva, et al. [21]	81	40	41	6	16

**Table 2:** Fitted random effect model

Estimate	Standard error	Z value	P-value	Lower bound	Upper bound
0.9324	0.4409	2.1148	0.0344	0.0683	1.7965

**Table 3:** Heterogeneity measure

Measure	Estimate	Lower Bound	Upper Bound
tau^2	0	0	>100.0000
tau	0	0	>10.0000
I^2(%)	0	0	>97.9720
H^2	1	1	>49.3097

Here, the estimated average log relative risk is equal to ˆμ=0.9324 (95% CI: 0.0683 to 1.7965). For easier interpretation, it may be useful to transform these values back to the relative risk scale through exponentiation (i.e., exp(ˆμ) = 2.54 with 95% CI: 1.07 to 6.03). The interpretation of these results, therefore, suggests that the risk of mortality in HCQ treated individuals is on average 2.5 times greater than in non-HCQ individuals. The null hypothesis H0: μ= 0 can be clearly rejected (p < 0.05).

**Figure 2:**
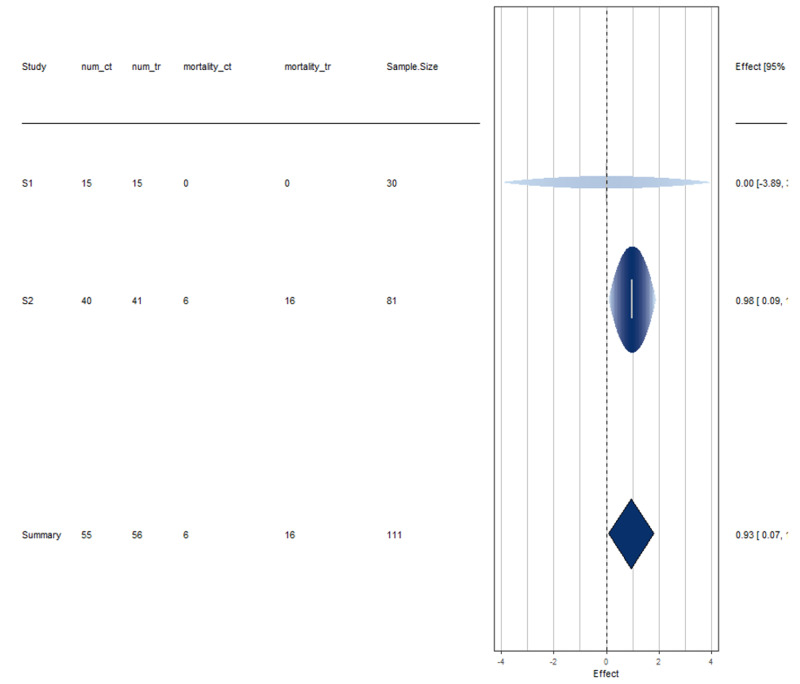
Disease mortality forest plot Interpretation Study 1 has more uncertainty in its results as evident due to the wide spread of the horizontal line. Studies 1 and 2 both do not cross the effect line at 0, indicating that they are not in agreement with the mortality rate of HCQ treated COVID-19 positive patients.

**Figure 3:**
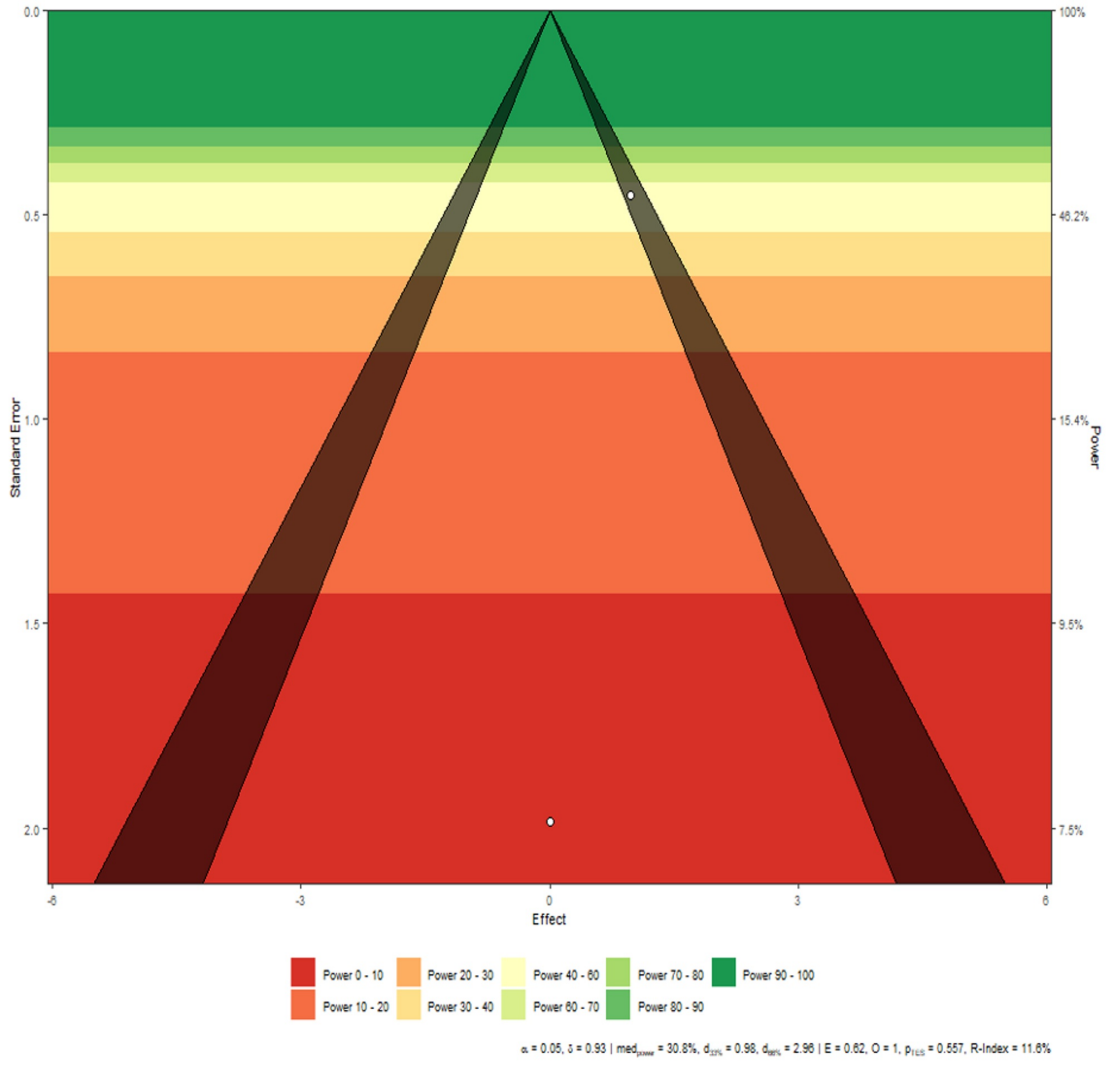
Disease mortality funnel plot Interpretation There is a marginal asymmetry between the studies, however, as the number of studies is small, this result can be attributed purely to chance rather than any actual publication bias.

These studies are perfectly homogeneous as tau^2 is 0 (equivalently, H^2 is 1). See Figures 2-3 for more information.

Section B: meta-analysis on progression/improvement rates

Among the six studies considered for meta-analysis, information on disease progression rates are available in four of them, details of which are provided below (Tables 4-6).

**Table 4:** Studies used in meta-analysis of improvement/progression rate

Name	Author	Sample Size	num_ control	num_ treatment	progression_ control	progression_ treatment
A pilot study of hydroxychloroquine in the treatment of patients with common coronavirus disease-19 (COVID 19)	Chen J, et al. [20]	30	15	15	7	5
Efficacy of hydroxychloroquine in patients with COVID-19 results of a randomized clinical trial	Chen et al. [22]	62	31	31	17	25
Treating COVID-19 with Chloroquine	Huang, et al. [23]	22	12	10	11	10
Hydroxychloroquine and azithromycin as a treatment of COVID-19 results of an open-label non- randomized clinical trial	Gautret, et al. [17]	36	16	20	0	20

**Table 5:** Fitted Random Effect Model

Estimate	Standard error	Z value	P-value	Lower bound	Upper bound
0.1839	0.2302	0.7987	0.4245	-0.2673	0.6351

**Table 6:** Heterogeneity measure

Measure	Estimate	Lower bound	Upper bound
tau^2	0.0495	0	48.0704
tau	0.2224	0	6.9333
I^2(%)	22.4095	0	99.6449
H^2	1.2888	1	281.598

Here, the estimated average log relative risk is equal to ˆμ=0.1839 (95% CI: -0.2673 to 0.6351) [4]. For easier interpretation, these values were transformed back to the relative risk scale through exponentiation (i.e., exp(ˆμ) = 1.2019 with 95% CI: 0.77 to 1.89). The interpretation of the results suggests that the disease progression rate in HCQ treated individuals is on average 1.2 times as large as the non-HCQ individuals. The null hypothesis H0: μ= 0 cannot be rejected (p > 0.05).

**Figure 4:**
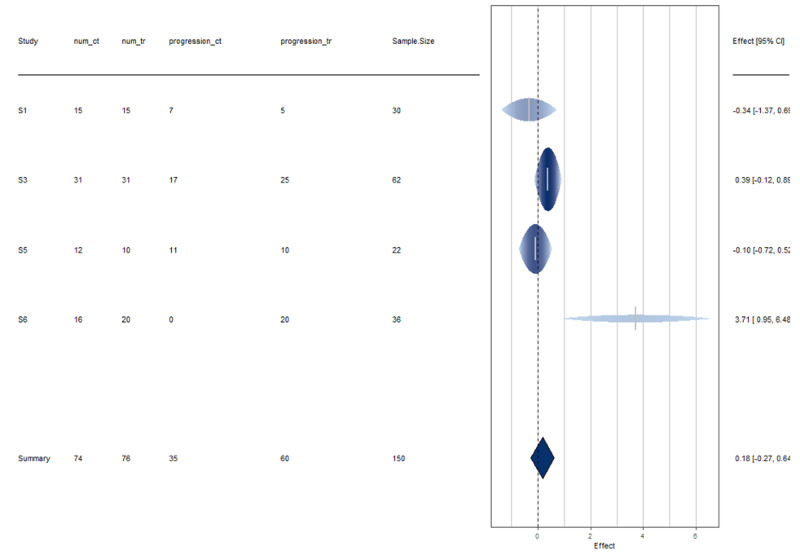
Disease progression forest plot Interpretation Study 6 has more uncertainty in its results as evident by the width of the horizontal line [19]. All studies, except Study 6, are in agreement with the results of a disease progression rate of HCQ treatment in patients with COVID [19].

**Figure 5:**
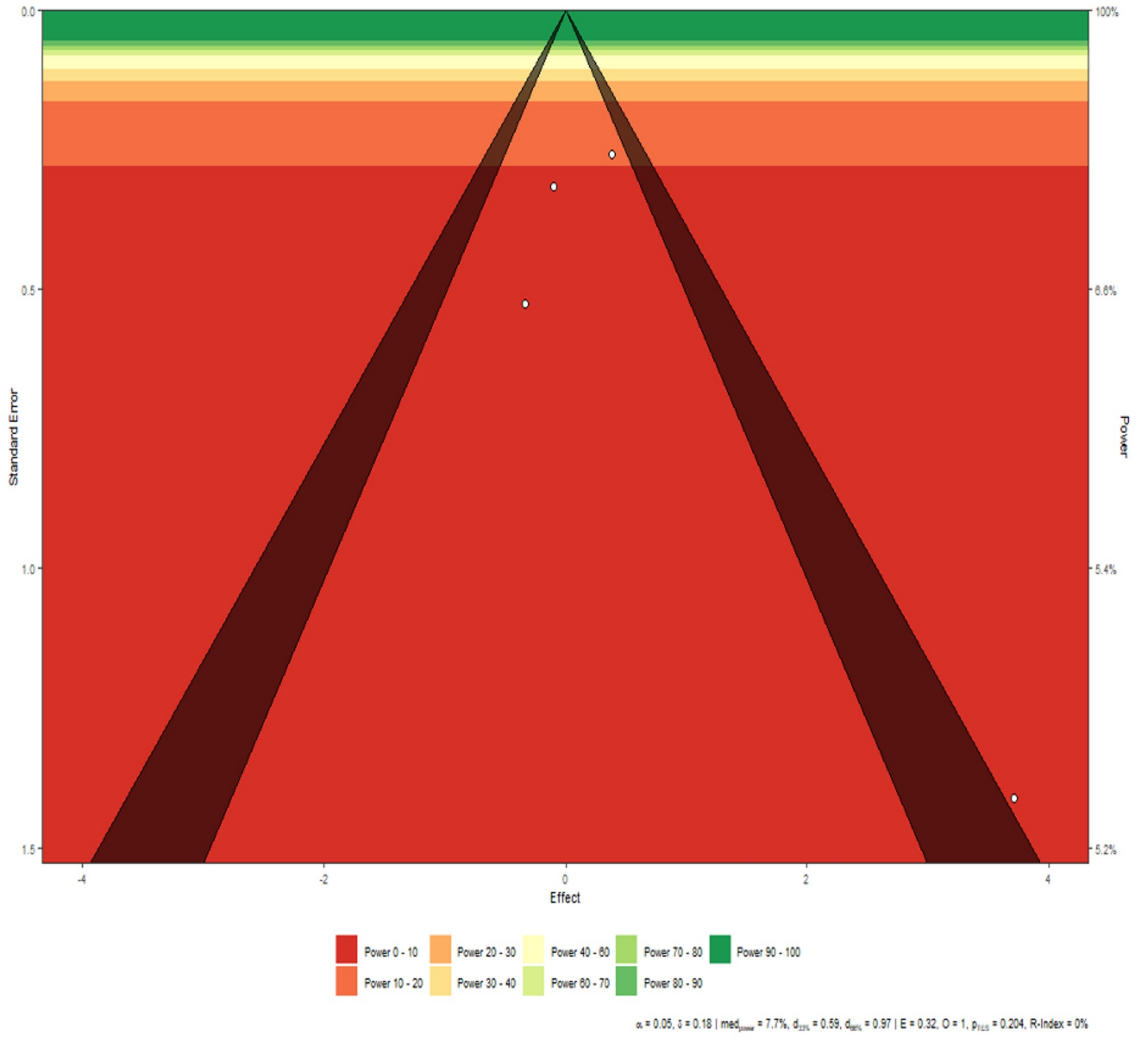
Disease progression funnel plot Interpretation The studies are symmetric except Study 6, which falls outside the triangle. This is in line with the conclusion drawn from the funnel plot. However, as evidenced by the funnel plot, Study 6 has very low power and thus its effect can be ignored.

These studies are a bit heterogeneous, though by a very small amount. See Figures 4-5 for more information.

Section C: meta-analysis on severity rates

Among the six studies considered, information on disease severity rates are available in four, the details of which are provided below (Tables 7-9).

**Table 7:** Studies used in meta-analysis of severity rate

Name	Author	Sample Size	num_ control	num_ treatment	severe_or adverse_control	severe_or adverse_treatment
A pilot study of hydroxychloroquine in the treatment of patients with common coronavirus disease 19 (COVID-19)	Chen J, et al. [20]	30	15	15	0	1
Hydroxychloroquine in patients with COVID-19: an open-label, randomized, controlled trial	Tang, et al. [24]	150	80	70	7	21
Treating COVID-19 with chloroquine	Huang, et al. [23]	22	12	10	5	O
Hydroxychloroquine: small effects in mild disease	Levantovsky, et al. [25]	62	31	31	4	0

**Table 8:** Fitted random effect model

Estimate	Standard error	Z value	P-value	Lower bound	Upper bound
-0.3644	0.9996	-0.3645	0.7155	-2.3236	1.5949

**Table 9:** Heterogeneity measure

Measure	Estimate	Lower bound	Upper bound
tau^2	2.4987	0	51.7919
tau	1.5807	0	7.1967
I^2(%)	65.7314	0	97.5465
H^2	2.9181	1	40.7582

Here, the estimated average log relative risk is equal to ˆμ=-0.3644 (95% CI: -2.3236 to 1.5949) [19]. For easier interpretation, these values are transformed back to the relative risk scale through exponentiation (i.e., exp(ˆμ) = 0.6946 with 95% CI: 0.10 to 4.92). The interpretation of these results suggests that the disease severity rate in HCQ treated individuals is on average 0.69 that of the non-HCQ individuals. The null hypothesis H0: μ= 0 can be rejected (p < 0.05).

**Figure 6:**
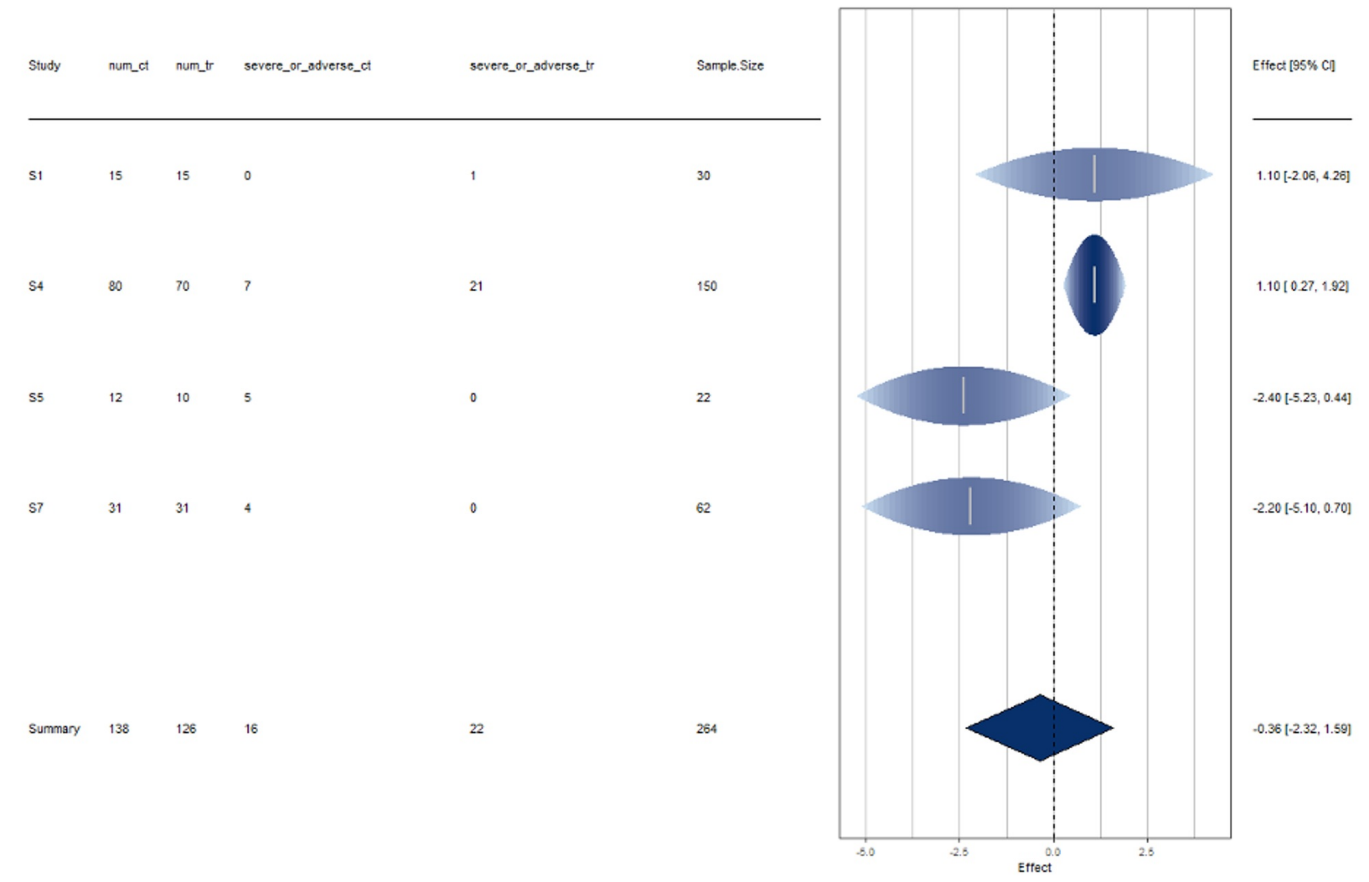
Disease severity forest plot Interpretation The results of all studies agree with the disease severity rate of HCQ treated COVID positive patients. There is slight disagreement shown by Study 4 by a moderate amount.

**Figure 7:**
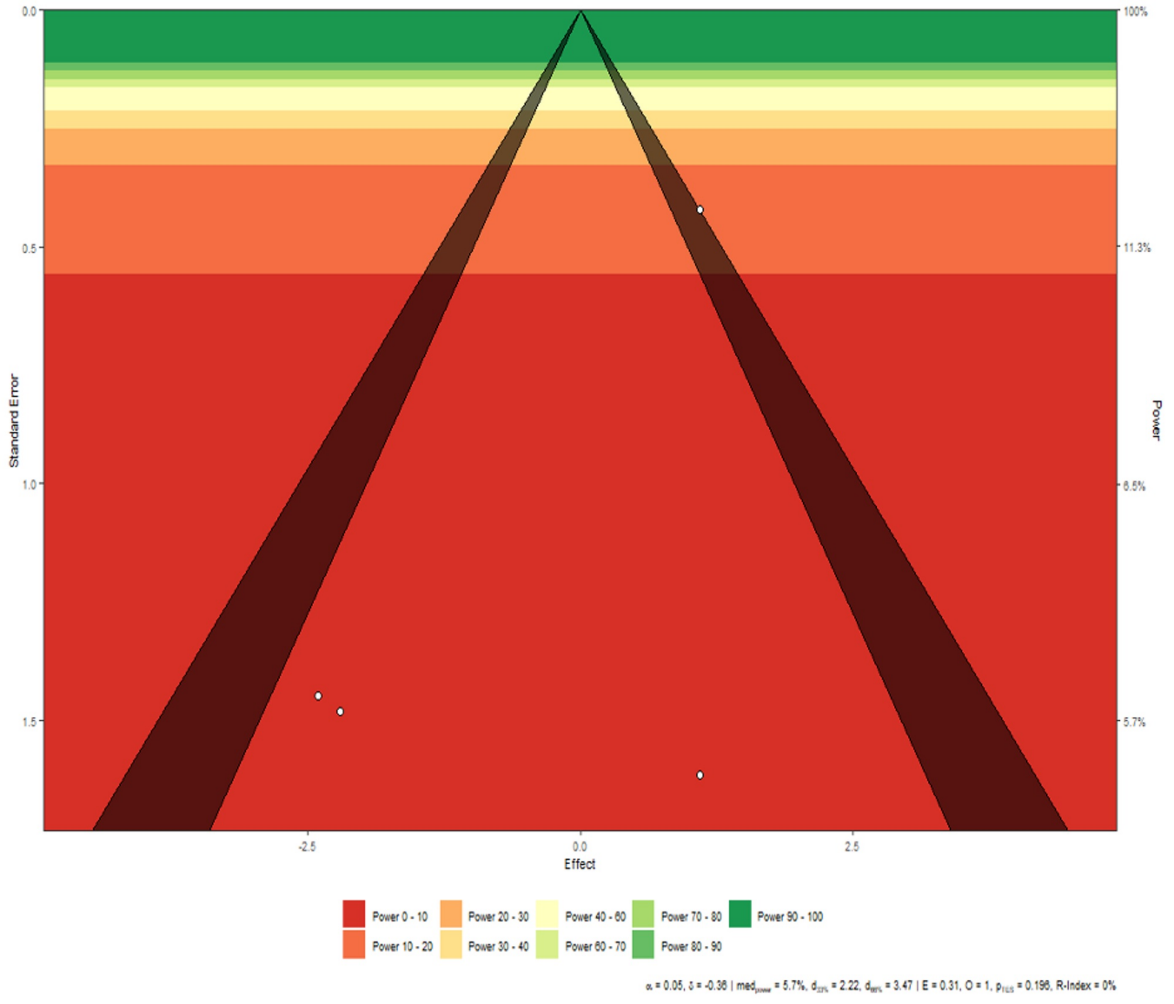
Disease severity funnel plot Interpretation The studies are symmetric except for Study 1, which falls on the border of the triangle. The remaining three studies, however, show symmetry among them.

These studies exhibit heterogeneity by a moderate amount. See Figures 6-7 for more information.

 

A summary of the meta-analyses' findings is provided in Table 10.

**Table 10:** Summary table

Meta-analysis on	# Studies used	Random effect model conclusion	Heterogeneity between studies	Conclusion from the forest plot	Conclusion from the funnel plot
Progression/ Improvement Rate	4	Progression rate in HCQ treated individuals is on average 1.2 times than non-HCQ individuals. This implies HCQ patients are slightly at an advantage on progression rate as compared to non-HCQ patients.	A bit of heterogeneity is observed among studies, which might be attributed to Study 6.	All studies are in agreement except Study 6, which shows some uncertainty in its results.	Study 6 has a bit of asymmetry, implying a small publication bias. It should be noted that study 6 has very low power also to distinguish the effect of HCQ from non-HCQ treatment.
Severity Rate	4	The severity rate in HCQ treated individuals is on average 0.69 times the non HCQ individuals. This means HCQ treated patients are 0.69 times more likely to face severe situations than non-HCQ patients.	Slight heterogeneity observed among studies	All studies are in agreement with respect to the conclusion.	Studies are symmetric except Study 1, showing slight asymmetry ie publication bias.
Mortality Rate	2	The risk of mortality in HCQ treated individuals is on average 2.54 times more than the non HCQ individuals.	No heterogeneity found, i.e., studies are homogeneous	Study 2 is more confident about its results and Study 1 is relatively less confident.	Slight asymmetry is noticed, however, as the number of studies are only two, it can be due to random effects.

## Discussion

The side effects of 4-Aminoquinolones are known to be dose-dependent increased risks for retinopathy, methemoglobinemia, and gastrointestinal (GI), renal, and cardiac toxicity [26]. HCQ co-administered with medications such as AZT further increases the risk of toxicity, particularly prolongation of the QT interval on electrocardiogram. The Borba et al. study revealed that males aged 50 with severe COVID symptoms and heart disease are at high risk for developing HCQ-related cardiac complications such as QT prolongation at higher doses of HCQ [21]. This toxicity is especially noted when combined with AZT, which is known to prolong the QT interval in populations with cardiac disease [21]. The studies by Tang et al. [24] and Chen J et al. [20] showed greater HCQ-related GI side effects as well.

In a post-marketing study by the Food and Drug Administration (FDA), it was also shown that the use of 4-Aminoquinolones increased rates of cardiac arrhythmias, ventricular tachycardia, fibrillation, and torsades de pointes. Their analysis also noted adverse cardiac events in combination with the use of other QT-prolonging medications such as azithromycin [27]. As a result, the FDA has cautioned the use of HCQ in COVID-19 patients, especially outside of the inpatient hospital setting [27]. Similarly, this meta-analysis supports that HCQ treated patients are more likely to have adverse side effects. It also appears that treatment with HCQ has a fatality rate of approximately 2.5 higher than with the control group.

The non-randomized study performed by Gautret et al. in the South of France included a total of 36 young patients with positive PCR test results and milder COVID-19 disease with no advanced comorbid medical conditions. A 50% reduction in viral load was noted at one week with a low dose of HCQ with AZT [17]. This study was not powered to detect mortality outcomes. Similarly, Yang et al. [19], Mingxing et al. [23], and Chen J et al. [20] studied females with a median age of 45 and mild COVID-19 related upper respiratory/pneumonia symptoms, without co-existing co-morbid medical disease. Patients were stated to have improved time to clinical resolution in the HCQ treatment arm [20,22-23]. These results seem to be in line with the meta-analysis’ of a slight disease improvement in COVID-19 patients treated with HCQ as compared with the controls.

Furthermore, recent studies show a gender disparity, in that females show better outcomes as compared to similar male cohorts [14]. This gender disparity is seen in a recent study that noted that male patients with advanced age or multiple comorbid medical conditions are at higher risk for mortality [11,14]. The studies in this meta-analysis did not include these high-risk patients with underlying complex co-morbid medical conditions, severe cases of COVID-19, ARDS, or critical care patient populations.

Limitations

Of note, the studies included in this meta-analysis have various definitions of control groups, which might affect the conclusion. However, with respect to the disease progression and severity meta-analysis, it appears that most of the studies are in agreement with the results, with slight exceptions, which might be attributed to chance. To get a more robust conclusion, the meta-analysis can be performed on more studies rather than six prospective control trials. Currently, there are 107 HCQ clinical trials in the active recruitment phase [28]; as the pandemic continues to unfold, these future large multicenter randomized controlled clinical trials may be included in the meta-analysis to conclude the size effect of HCQ on COVID-19.

## Conclusions

Our study looks at three disease outcome measures of treatment with HCQ in patients with COVID-19: mortality rates, progression rates, and severity rates. In terms of mortality rates, it appears treatment with HCQ has a fatality rate that is 2.5 times greater than that of the control group. Similarly, HCQ treated patients are more likely to have an adverse clinical outcome and side effects. Lastly, there was a 1.2-times higher rate of clinical improvement in the group of HCQ treated patients, with mild to moderate symptoms as compared to the control group.
